# The assessment of serum trace element levels as the diagnostic biomarkers of functional state of broiler chickens

**DOI:** 10.14202/vetworld.2023.1512-1519

**Published:** 2023-07-24

**Authors:** Svyatoslav Lebedev, Tatiana Kazakova, Olga Marshinskaia, Victoria Grechkina

**Affiliations:** 1 Federal Research Centre of Biological Systems and Agrotechnologies of the Russian Academy of Sciences, 460000, Orenburg, Russia; 2 Department of the Non-Communicable Diseases of Animals, Orenburg State Agrarian University, 460014, Orenburg, Russia

**Keywords:** blood biochemistry, blood serum, broiler chickens, feeding, trace elements

## Abstract

**Background and Aim:**

Elemental analysis is a useful technique for predicting metabolic disorders in broiler chickens. Elemental imbalances are also important for the development of new methods to diagnose the health status of birds that can be implemented on a farm-wide scale. This study aimed to identify elemental markers related to pre-nosological diagnoses of metabolic disorders in broiler chickens.

**Materials and Methods:**

We compared birds given high-protein, high-carbohydrate, and high-fat diets. A control group received the standard diet recommended by the All-Russian Research and Technological Institute of Poultry, while experimental Group I received a diet with high-protein content, Group II received a diet with high-carbohydrate content, and Group III received a diet with high-fat content. At the end of the experiment, blood samples were taken for biochemical and elemental analysis. Biochemical analysis was carried out using an automated biochemical analyzer, and the levels of trace elements in the serum were assessed using inductively coupled plasma spectrometry.

**Results:**

We found that the high-protein diet was accompanied by a decrease in chicken body weight, cholesterol, and several elements (i.e., P, Cr, Cu, Zn, and B) as well as an increase in the levels of Ca, Co, and Si. The high-carbohydrate diet led to a significant increase in glucose levels as well as a decrease in the levels of albumin, triglycerides, and Cr, Mn, Se, I, and Cu. Finally, the high-fat diet led to an increase in body weight, glucose, cholesterol, triglycerides, and the elements Cu, Zn, and Si as well as a decrease in the levels of Mg, Cr, and Fe.

**Conclusion:**

The determination of the levels of trace elements such as Co, Cr, Mn, Fe, and Cu in chicken blood serum may be an important indicator of the state of protein, carbohydrate, and lipid metabolism of poultry stock.

## Introduction

Poultry farming is one of the largest segments of the global agricultural sector [1, 2]. Over the past 60 years, broiler chicken production has significantly improved; for example, the live weight of broilers at ages of 33–35 days can now reach as high as 2.6 kg. Such improvements are due to the achievements of modern genetics, breeding, and improving technologies associated with chicken growth and feeding [3]. According to the Food and Agriculture Organization of the United Nations, poultry production may reach 181 million tons by 2050 [4, 5].

In the future, broiler production will become more competitive by developing new approaches to achieve higher productivity, increase product quality, and reduce environmental impacts [6–8]. However, introducing new technologies can introduce stress to the metabolic systems of birds. This can lead to various diseases, especially metabolic pathologies [9]. However, the previous research has found that only physiologically healthy birds can maximize their biological or genetic potential.

Under such conditions, the early diagnosis of poultry diseases is critically important since timely detection of metabolic disorders at the stage of preclinical manifestation facilitates the implementation of necessary therapeutic and preventive measures, including adjustments to the feeding and keeping of poultry. The activity of physiological processes occurring with the bodies of the birds is most fully characterized by the level of chemical elements in the blood, the content of which can be used to judge metabolic states and the degree of adaptive reactivity [10]. This is justified because most macro- and trace elements are integral parts of various enzyme systems across the body. Furthermore, studies have established that macro- and microelements directly impact protein, carbohydrate, and lipid metabolism, which is why the elemental system is a fundamental regulator of all bodily functions [11]. Data characterizing elemental flow are therefore important for developing new methods for screening bird health that can be further applied on farms.

This study aimed to identify elemental markers for the pre-nosological diagnosis of metabolic disorders in the bodies of broiler chickens.

## Materials and Methods

### Ethical approval

The experimental studies were carried out in accordance with the instructions and recommendations of the Russian regulations (Order of the Ministry of Health of the USSR No.755 of August 12, 1977 “On measures to further improve the organizational forms of work using experimental animals”), the protocols of the Geneva Convention, and the principles of good laboratory practice (National Standard of the Russian Federation GOST R 53434-2009). All procedures on animals were performed in accordance with the rules of the Animal Ethics Committee of the FRC BST RAS.

### Study period and location

This study was conducted from May to July 2022. The study was conducted at the Federal State Budgetary Scientific Institution “Federal Scientific Center for Biological Systems and Agrotechnologies of the Russian Academy of Sciences” (accreditation certificate of the State Standard of Russia - PA.RU21ПФ59 dated December 2, 2015).

### Experimental animals

Arbor Icres cross (CJSC “Orenburgskaya Poultry Farm”) broiler chickens (n = 60) were used. After an initial post-hatching preparatory period of 7 days, broiler chickens were divided into four groups of 30 birds each. The control group received a balanced dry matter (DM) diet that followed the recommendations of the All-Russian Research and Technological Institute of Poultry [12]. In contrast, the differences in the diets of the experimental groups were as follows: Group I birds received a protein-enriched DM diet that included 10% casein, Group II birds received a carbohydrate-enriched DM diet that included 10% dextrose, and Group III birds received a fat-enriched DM diet that included 10% sunflower oil. All chickens were given food and water *ad libitum*. Modeling of diets was carried out in the framework of the research under Project No. 21-16-00009 with the support of the Russian Science Foundation.

According to the nutritional recommendations, broiler chickens were reared on a three-phase diet throughout the experimental period. A starter diet was provided from days 0–10, a grower diet from days 11–20, and a finishing diet from days 21–35. The feed composition included the following ingredients: wheat, barley, corn, soybeans, soybean meal, sunflower meal, sunflower oil, limestone flour, table salt, meat meal, amino acids, vitamins, and mineral premix (Table-1).

**Table-1:** Composition of the diet.

Component	Starter feed	Grower feed	Finisher feed
NE, kcal/100 g	305	307	311
Crude protein, %	23.0	21.7	18.0
Crude fat, %	5.15	5.99	6.48
Crude fiber, %	3.55	3.73	4.77
DM, %	89.29	89.29	91.57
Lysine, %	1.43	1.32	1.08
Methionine+cysteine, %	1.08	1.01	0.86
Threonine, %	0.98	0.9	0.77
Ca, %	1.0	0.91	0.91
P, %	0.83	0.82	0.74
K, %	0.76	0.71	0.6
Na, %	0.17	0.2	0.15
Biologically active substances			
Vitamin А, KIU/kg	14.4	12.0	12.0
Vitamin D3, KIU/kg	4.8	4.0	4.0
Vitamin E, mg/kg	72.0	60.0	60.0
Vitamin K3, mg/kg	2.4	2.0	2.0
Vitamin B1, mg/kg	2.4	2.0	2.0
Vitamin B2, mg/kg	9.6	8.0	8.0
Vitamin B3, mg/kg	36.0	30.0	30.0
Vitamin B4, mg/kg	600.0	500.0	500.0
Vitamin B5, mg/kg	12.0	10.0	10.0
Vitamin B6, mg/kg	3.6	3.0	3.0
Vitamin B12, mg/kg	0.03	0.025	0.025
Vitamin B9, mg/kg	0.6	0.5	0.5
Vitamin H, mg/kg	0.12	0.1	0.1
Fe, mg/kg	30.0	25.0	25.0
Cu, mg/kg	12.0	10.0	10.0
Zn, mg/kg	96.0	80.0	80.0
Mn, mg/kg	96.0	0	80.0
Co, mg/kg	1.2	0	1.0
I, mg/kg	0.84	0	0.7

DM=Dry matter, NS=Net energy

The duration of the experiment was 28 days. Birds were maintained in KB-20-2 cages (https://profifermer.ru). The temperature regime and relative humidity corresponded to the norms recommended for growing broilers. The photoperiod program complied with European Social Security Regulation 43/2007 (i.e., Council Directive 2007/43/EU, which provides minimal animal welfare rules to protect chickens kept for meat production).

### Assessment of growth indicators

The dynamics of growth rates were assessed by weighing each bird before feeding on the 7^th^, 14^th^, 21^st^, 28^th^, and 35^th^ days of the experiment. An average daily mass gain was calculated using these results. The feed conversion ratio was calculated on the 35^th^ day of the experiment. Mortality was recorded as it occurred and the general health of each bird was monitored throughout the experimental period.

### Sampling and preparation of blood samples

Blood sampling of all birds was carried out through extraction from the axillary vein on the morning of the 35^th^ day of the experiment. Vacuum tubes with a blood-clotting activator and a gel for separating erythrocyte mass were used for all blood samples (Greiner Bio-One International AG, Austria). Eppendorf tubes with serum were then subjected to low-temperature freezing (–70°C) and stored in an 803CV freezer (Thermo Fisher Scientific, Germany) until further analysis, and only serum samples without signs of hemolysis were used for downstream analyses.

### Laboratory tests

#### Biochemical analysis of blood serum

Serum analyses were carried out on CS-T240 automatic biochemical analyzer (Dirui Industrial Co., Ltd, China) using Randox commercial biochemical kits (USA). Biochemical determinations were performed for glucose, total protein, albumin, total cholesterol, and triglyceride content. All procedures were carried out at the Central Collective Use Center of the FSSI FRC BST RAS.

#### Elemental analysis of blood serum

For elemental analysis, serum samples were first diluted to 1:15 (w/w) with an acidified (pH = 2.0) diluent consisting (w/w) of 1% 1-butanol (Merck KGaA, Darmstadt, Germany), 0.1% Triton X-100 (Sigma-Aldrich, Co., St. Louis, USA), and 0.07% HNO_3_ (Sigma-Aldrich, Co.) in distilled deionized water (18 MOM cm^−1^) (Merck Millipore, Billerica, Massachusetts, USA). Next, the macronutrient (i.e., Ca, K, Mg, Na, and P), vital microelement (i.e., Co, Cr, Cu, Fe, I, Li, Mn, Se, Si, V, and Zn), and toxic microelement (i.e., As, B, Cd, Hg, Ni, Pb, Sn, and Sr) contents of all samples were determined using a NexION 300D spectrometer (Perkin Elmer, USA). The elemental content of broiler blood samples was assessed within the framework of research project No. 22-16-00070 supported by the Russian Science Foundation.

### Statistical analysis

All data were analyzed using Statistica version 10 (StatSoft Inc., USA), and original data were stored and preprocessed using Microsoft Excel 2010 (Microsoft, USA). The normality of the obtained data was checked using Shapiro–Wilk tests. The hypothesis that the data belonged to a normal distribution was rejected in all cases with a probability of 95%, which justified the use of non-parametric procedures for downstream statistical analyses; we therefore examined differences among group means using Mann–Whitney U-tests. The data obtained were presented as a median (Me) and as 25^th^–75^th^ percentiles (Q_25_–Q_75_). For all statistical analyses, the achieved level of significance (p) was calculated, and the critical level of significance in this study was taken to be ≤0.05. Relationships between parameters were assessed using Spearman rank correlations. To determine the strength of the relationship between the studied variables, we calculated correlation coefficients (r). These were interpreted as follows: <0.3: Weak correlation; 0.3 < r < 0.5: Moderate correlation, 0.5 > r > 0.7: Significant correlation, 0.7 > r > 0.9: Strong correlation, and >0.9: Very strong correlation.

## Results

### Assessment of growth and feed intake

For the first (protein) experimental group, we found that broiler live weight was below the control values throughout the experiment. However, statistically significant differences were found only on the 21^st^ and 35^th^ days of the experiment; here median bird live weight was lower by more than 5% (p ≤ 0.05) and 13% (p ≤ 0.05) relative to the control, respectively. We note that the average daily gain of the birds of the first experimental group was statistically significantly lower than the control by 14% (p ≤ 0.01) (Table-2). In broiler chickens from the second (carbohydrate) experimental group, the live weight during the first weeks of the experiment did not differ significantly from the control. However, at the end of the study, we observed lower values for median body weight and average daily gain. In the birds from the third (fat) experimental group, the most significant changes in weight were noted on the 14^th^ day of the experiment, where we observed that bird weight was significantly higher than control values by 8% (p ≤ 0.03). We also found an increase of 4% on day 21 (p ≤ 0.002) and by 12% by day 35 (p ≤ 0.05). The average daily increase was also 13% higher than the control (p ≤ 0.05).

**Table-2:** Physiological indicators of broiler chickens with the different nutrient supply of the diet, g.

Days	Control group	I experimental group	II experimental group	III experimental group
Body weight, g				
7	186.1 (180.2–188.5)	186.0 (176.7–189.6)	180.0 (175.0–182.6)	183.0 (181.8–186.5)
14	451.3 (448.5–456.5)	445.3 (421.5–458.5)	452.0 (451.2–458.0)	485.7 (479.0–489.5)^c^
21	862.3 (855.0–866.5)	815.1 (801.7–835.5)^a^	863.1 (858.0–864.4)	895.2 (893.7–895.9)^cc^
28	1447.7 (1429.1–1487.0)	1161.0 (1057.1–1265.7)	1415.0 (1413.0–1417.0)	1509.5 (1500.0–1516.6)
35	2202.0 (2109.5–2210.5)	1922.2 (1860.5–2174.1)^a^	2107.5 (2104.0–2109.5)	2467.3 (2292.5–2522.0)^c^
Daily weight gain, g/day				
7–35	72.0 (68.8–72.2)	62.1 (58.4–70.04)^aa^	68,9 (68,7–69,0)	81.2 (75.2–83.5)^c^
Feed intake, g/bird				
7–35	4441.0 (4105.5–4907.6)	4115.2 (4001.0–4457.5)	4224.7 (4988.0–4333.6)	4003.5 (4789.2–4149.5)^c^
Feed conversion ratio				
35	2.2 (1.9–2.3)	2.27 (2.0–2.3)	2.2 (2.0–2.3)	1.75 (1.6–2.0)^c^

a (p ≤ 0.05);

aa (p ≤ 0.01) p-level comparing experimental group I with control group; ^b^(p ≤ 0.05); ^bb^(p ≤ 0.01) =p-level comparing experimental group II with control group;

c (p ≤ 0.05);

cc (p ≤ 0.01))=p-level comparing experimental group III with control group

In experimental Groups I and II, we also found that birds showed a decrease in the amount of food consumed over the course of the experiment. However, in birds from the third experimental group, the level of feed consumption was significantly lower than the control by 10% (p ≤ 0.04). It should be noted that the feed conversion coefficient was statistically significantly decreased only in experimental Group III.

### Blood chemistry analyses

Next, biochemical blood analyses revealed that the inclusion of additional casein in the diet of birds contributed to a decrease in cholesterol in the blood serum (Table-3). Moreover, this reduction was 14% (p = 0.05) lower than the control and was accompanied by a reduction in triglyceride content. Furthermore, broilers supplemented with a high-sucrose diet showed a statistically significant increase in blood glucose of 7% (p = 0.02) as well as a decrease in albumin and triglyceride levels. Finally, consumption of a high-fat diet resulted in a 4% increase in glucose (p = 0.05), a 3% increase in cholesterol (p = 0.01), and a 60% increase in triglycerides (p = 0.03).

**Table-3:** Biochemical parameters of the blood of broiler chickens with the different nutrient supply of the diet.

Indicators	Control group	I experimental group	II experimental group	III experimental group
Glucose, mmol/L	11.57 (10.03–11.87)	11.96 (11.02–12.2)	12.36 (11.98–14.04)^b^	12.09 (11.89–13.5)^c^
Total protein, g/L	30.4 (28.3–32.8)	29.89 (26.01–33.3)	29.08 (27.02–31.2)	27.6 (26.0–29.4)
Albumin, g/L	16.0 (14.6–17.5)	16.04 (13.9–17.9)	14.33 (12.2–15.8)	13.34 (12.8–15.1)
Cholesterol, mmol/L	3.36 (3.1–3.89)	2.89 (2,7–3.1)^а^	3.3 (2.9–3.7)	3.46 (3.23–3.9)^сс^
Triglycerides, mmol/L	0.35 (0.27–0.54)	0.25 (0.21–0.33)	0.31 (0.25–0.44)	0.56 (0.48–0.66)^с^

^a^(p ≤ 0.05)=p-level comparing experimental group I with control group;

* ^b^(p ≤ 0.05) =p-level comparing experimental group II with control group;

c (p ≤ 0.05); ^cc^(p ≤ 0.01)= p-level comparing experimental group III with control group

### Elemental composition of blood samples

Next, we performed elemental analyses of broiler blood serum samples and found that by the 35^th^ day of the experiment, broilers in experimental Group I showed changes in mineral metabolism. Specifically, we found a significant increase in the levels of Ca, Co, and Si by 22% (p = 0.01), 217% (p = 0.003), and 60% (p= 0.05), respectively, relative to the control. Against this background, we also observed a decrease in the content of a number of macro and microelements. For example, P was reduced by 44% (p = 0.01), Cr by 18% (p = 0.02), Zn by 5% (p = 0.02), Cu by 14% (p = 0.05), and B by 17% relative to the control (p = 0.02; Table-4). In the second experimental group, changes in calcium-phosphorus metabolism, including a statistically significant increase in Ca levels by 14% (p = 0.003) and a 26% decrease in P (p = 0.02). We also found a significant decrease in essential microelement content, including a reduction in Cr by 21% (p = 0.05), Cu by 18% (p = 0.002), I by 34% (p = 0.01), Mn by 84% (p = 0.02), and Se by 21% (p = 0.03) compared to the control.

**Table-4:** Elemental composition of blood serum of broiler chickens with the different nutrient supply of the diet.

Indicators	Control group	I experimental group	II experimental group	III experimental group
Macroelements				
Ca	0.117 (0.11–0.124)	0.143 (0.138–0.144)^aa^	0.133 (0.129–0.138)^bb^	0.12 (0.118–0.122)
K	0.257 (0.242–0.265)	0.22 (0.205–0.221)	0.215 (0.212–0.218)	0.233 (0.228–0.236)
Mg	0.0294 (0.0292–0.0297)	0.0285 (0.0276–0.029)	0.0287 (0.0284–0.0292)	0.0187 (0.0185–0.0189)^сс^
Na	3.01 (2.63–3.08)	2.9 (2.79–3.06)	3.04 (3.0–3.08)	3.03 (2.78–3.06)
P	0.249 (0.24–0.249)	0.139 (0.135–0.147)^аа^	0.183 (0.18–0.191)^b^	0.2 (0.185–0.209)
Vital and conditionally vital trace elements				
Co	0.0017 (0.0014–0.0018)	0.0054 (0.005–0.0056)^aa^	0.0027 (0.0025–0.0029)	0.0035 (0.0033–0.0037)
Cr	0.0086 (0.0083–0.0091)	0.0069 (0.0063–0.0074)^а^	0.0068 (0.0064–0.008) ^b^	0.0058 (0.0045–0.0061)^с^
Cu	0.143 (0.139–0.146)	0.124 (0.105–0.128)^а^	0.117 (0.113–0.118)^bb^	0.161 (0.154–0.162) ^cc^
Fe	1.531 (1.495–1.558)	1.67 (1.634–1.7)	1.655 (1.621–1.675)	1.36 (1.259–1.4)^сс^
I	0.05 (0.046–0.053)	0.062 (0.0603–0.064)	0.033 (0.03–0.035)^bb^	0.018 (0.016–0.018)
Mn	0.0092 (0.0088–0.0092)	0.0088 (0.0077–0.0089)	0.0014 (0.0012–0.0015)^bb^	0.008 (0.007–0.0086)
Se	0.188 (0.178–0.189)	0.174 (0.162–0.178)	0.148 (0.133–0.154)^b^	0.176 (0.169–0.178)
Zn	2.19 (2.13–2.22)	2.07 (1.98–2.11)^а^	2.49 (2.4–2.55)	2.59 (2.34–2.66)^c^
B	0.46 (0.43–0.47)	0,38 (0,37–0,39)^а^	0.44 (0.4–0.46)	0.36 (0.33–0.38)
Ni	0.0071 (0.0069–0.0074)	0.006 (0.0054–0.007)	0.005 (0.0043–0.0054)	0.0078 (0.0071–0.0092)
V	0.0041 (0.0039–0.0043)	0.0044 (0.0035–0.0049)	0.0052 (0.0047–0.0054)	0.0051 (0.0049–0.0055)
Li	0.0182 (0.0169–0.0187)	0.0065 (0.0057–0.008)	0.0082 (0.0074–0.0083)	0.0184 (0.0178–0.0188)
Si	51.12 (50.44–52.62)	81.64 (78.81–85.35)^а^	78.36 (77.82–78.53)	93.57 (91.95–94.08)^c^
As	0.0033 (0.0031–0.0037)	0.0037 (0.0034–0.0039)	0.0034 (0.0031–0.0037)	0.0034 (0.0027–0.0036)
Toxic microelements				
Cd	<0.0009	<0.0009	<0.0009	<0.0009
Hg	<0.0001	<0.0001	<0.0001	<0.0001
Pb	<0.0005	<0.0005	<0.0005	<0.0005
Sn	<0.0005	<0.0005	<0.0005	<0.0005
Sr	0.13 (0.128–0,132)	0.119 (0.117–0.121)	0.119 (0.109–0.122)	0.081 (0.076–0.083)

^a^(p ≤ 0.05);

aa (p ≤ 0.01)=p-level comparing experimental group I with control group;

b (p ≤ 0.05);

bb (p ≤ 0.01)=p-level comparing experimental group II with control group;

c (p ≤ 0.05);

cc (p ≤ 0.01)=p-level comparing experimental group III with control group

In the third experimental group, we found a 36% of reduction in Mg content relative to the control (p = 0.003), as well as a reduction of Cr by 33% (p = 0.05) and of Fe by 11% (p = 0.01). In contrast, the serum Cu level was statistically significantly higher than the control group by 13% (p = 0.003), and Si and Zn showed respective increases of 83% (p = 0.003) and 18% (p = 0.05).

Next, we performed correlation analyses to identify “catalyzing elements” involved in protein, fat, and carbohydrate metabolism (Table-5). According to this analysis, we found that blood biochemical parameters correlated significantly with a number of macro- and microelements. In particular, the level of total protein was significantly associated with P (r = 0.8; p = 0.003), K (r = 0.78; p = 0.05), Co (r = 0.87; p = 0.05), B (r = 0.7; p = 0.01), and Li (r = 0.86; p = 0.02) in broiler blood serum sample from the first experimental group. In addition, differences in glucose values were statistically significantly associated with P (r = 0.7; p = 0.02), Cr (r = 0.8; p = 0.01), Mn (r = 0.77; p = 0.003), and Li (r = 0.73; p = 0.003) in broiler blood serum samples taken from the second experimental group. Finally, the total cholesterol level was found to be correlated with Fe (r = 0.88; p = 0.01) and Cu (r = 0.86; p = 0.01) in blood samples of broilers from the third experimental group.

**Table-5:** “Elements–catalysts” of protein, carbohydrate and fat metabolism.

Groups	I experimental group	II experimental group	III experimental group
Broiler chickens	P (r = 0.8; p *=* 0.003), K (r = 0.78; p *=* 0.05), Cо (r = 0.87; p *=* 0.05), B (r = 0.7; p *=* 0.01), Li (r = 0.86; p *=* 0.02)	P (r = 0.7; p *=* 0.02), Cr (r = 0.8; p *=* 0.01), Mn (r = 0.77; p *=* 0.003), Li (r = 0.73; p *=* 0.003)	Fe (r = 0.88; p *=* 0.01), Cu (r = 0.86; p *=* 0.01)

## Discussion

The results of this study demonstrate that increasing the protein content of the diet of broiler chickens had an adverse effect on broiler body weight and average daily gain in mass. Such measurements for Group I were below control values throughout the experiment, even as the amount of feed consumed declined. These data are consistent with previous studies by French *et al*. [13] that showed a decrease in the body weight of experimental animals in response to consumption of a high-protein diet. It is possible that a decrease in the amount of feed consumed is associated with an increase in broiler satiety. When assessing the biochemical parameters of blood from birds of the first experimental group, we found decreased cholesterol and triglyceride content. This may be associated with an additional load on the digestive system and on the functional activity of the liver [14]. We also identified specific changes in the mineral metabolism of birds, since Group I broilers showed a significant increase in the blood serum levels of Ca, Co, and Si but a decrease in the levels of P, Cr, Cu, Zn, and B. Moreover, statistically significant correlations were found between total protein content and P, K, Co, B, and Li levels in the blood. Thus, the data obtained show that changes occurred primarily related to phosphorus-calcium metabolism. According to the previous studies by Tang *et al*. [15], the consumption of high-protein diets can cause an increase in calcium absorption in the intestine, which results in an increase in the calcium concentration of the blood. Calcium, in turn, can act as an antagonist, making it difficult to assimilate a number of other macro and microelements, including phosphorus, magnesium, sodium, potassium, copper, and zinc. This is consistent with our findings, since we observed a significant decrease in Cu and Zn content [16]. Moreover, maintaining the balance of calcium and phosphorus levels is required for all vertebrates to ensure various biological processes, including bone formation, blood coagulation, cell proliferation, and the maintenance of energy metabolism [17]. However, researchers do not recommend the use of serum Ca and P values as parameters for assessing the elemental status of broilers [18]. This is because the blood concentration of these elements in broilers remains in a rather narrow physiological range, regardless of the different levels of Ca in the diet [19].

In broilers supplemented with extra protein, we observed large changes in cobalt content and found a strong correlation between cobalt content and total protein content. Cobalt is an important trace element because it is a cofactor of Vitamin B12 and is also necessary for the formation of amino acids and some proteins [20]. In addition, birds from Group I also showed decreased chromium content. Cr affects protein absorption and metabolism [21], and previous studies have found that chromium deficiency in animals leads to impaired incorporation of amino acids (e.g., glycine, serine, and methionine) into heart muscle. In addition, one study also noted increased uptake of amino acids and glucose by skeletal muscle in animals given a chromium picolinate dietary supplement. Overall, the observed improvement in amino acid incorporation was beneficial to overall protein deposition. Thus, our data show that broilers consuming a high-protein diet showed changes in mineral metabolism. However, this should be studied further, because violation of elemental homeostasis can trigger a cascade of important changes, some of which may decrease the growth rate and mineralization of bird bone tissue [19].

Next, we found that adding carbohydrates to broiler feed did not significantly affect key physiological parameters (e.g., body weight, average daily gain, and feed intake) of birds from the second experimental group. However, we still observed a tendency for the values of these indicators to be lower in Group II birds relative to control group birds. At the same time, the biochemical composition of the blood was characterized by a significant increase in glucose levels and a decrease in the levels of albumin and triglycerides. These results are consistent with studies conducted on other animal models [22]. Furthermore, elemental status of birds from Group II identified changes in calcium-phosphorus metabolism and a decrease in the content of a number of trace elements including Cr, Mn, Se, I, and Cu. We also found a strong association between glucose and the elements P, Cr, Mn, and Li. Thus, chromium is also necessary for maintaining normal carbohydrate metabolism, likely since it is an activator of the glucose tolerance factor responsible for increasing the efficiency of insulin metabolism [23]. Moreover, studies have reported that increased consumption of simple sugars results in increased chromium loss [24]. Manganese is an equally important trace element for the regulation of carbohydrate metabolism [25], and decreased manganese levels can contribute to several structural and physiological disorders, including decreased effectiveness of the antioxidant defense system, the formation of skeletal and cartilage malformations, and impaired reproductive function [26]. Finally, we also note that other studies have also found decreased levels of essential microelements in response to variations in carbohydrate metabolism [27].

Consumption of a high-fat diet resulted in increased bird weight and average daily gain while reducing feed intake and feed conversion rate. Despite the potential benefits of this diet, a detailed examination of the biochemical and elemental content of serum blood samples revealed a number of deviated metabolic processes in broilers from Group III. Consumption of the high-fat diet was associated with increases in glucose, cholesterol, and triglyceride content, a finding that is consistent with studies of laboratory animals. In one report, the authors found a significant increase in glucose and triglyceride levels in rats fed a diet containing 45% fat [28]. These data also clearly demonstrated changes in the macro and microelement content in the blood serum of broilers fed a high-fat diet. Serum analyses revealed a decrease in Mg, Cr, and Fe levels and a significant increase in Cu, Zn, and Si levels. Moreover, strong correlations were also found between total cholesterol, Cu, and Fe. According to previous studies by Rayssiguier *et al*. [29], Mg deficiency is often observed in response to altered lipid metabolism, and serum Mg levels have been reported to be strongly associated with metabolic risk factors in obesity [30]. It should be noted that a number of studies have also shown a decrease in blood serum iron concentration [31]. It is assumed that such changes may occur in connection with a chronic inflammatory response and the subsequent hyperproduction of hepcidin [32]. We also found increased serum copper levels in high-fat diet broilers, which is consistent with the results of previous studies by Yang *et al*. [33] that demonstrated a positive association between elevated serum copper and obesity. Finally, it has also been suggested that elevated serum Cu levels are associated with altered leptin and insulin levels.

Taken together, the results of the experiment show that disturbances in the mineral metabolism of broilers occur in birds given high-protein, high-carbohydrate, and high-fat diets. These can, in turn, lead to adverse consequences, including a decrease in productivity of the bird stock.

## Conclusion

This study showed that the supplementation of protein, carbohydrate, and fat nutrients into the diets of broiler chickens affected the course of key metabolic processes in the body. Biochemical and mineral stability is an essential condition for the normal functioning of the body, and consequently the results of the study show that there is a certain threshold, above and below which the protein, carbohydrate, and fat content becomes abnormal. When nutrients within the diet exceed these levels, a positive result with respect to growth, health, and other performance indicators is not expected. Therefore, based on the results obtained in this study, we conclude that the determination of blood serum Co, Cr, Mn, Fe, and Cu levels in broiler chickens has great potential as an indicator of the state of protein, carbohydrate, and lipid metabolism.

## Authors’ Contributions

TK, OM, ST, and VG: Study conception and design and revised the manuscript. TK, OM, and VG: Material preparation, data collection, and analysis. SL: Drafted the manuscript. All authors have read and approved the final manuscript.
